# Ewing’s sarcoma arising from the adrenal gland in a young male: a case report

**DOI:** 10.1186/1756-0500-6-533

**Published:** 2013-12-13

**Authors:** Muhammad Nauman Zahir, Tayyaba Zehra Ansari, Tariq Moatter, Wasim Memon, Shahid Pervez

**Affiliations:** 1 Department of Oncology, Aga Khan University Hospital, Stadium Road, PO BOX: 3500, Karachi 74800, Pakistan; 2 Department of Pathology and Microbiology, Aga Khan University, Stadium Road, PO BOX: 3500, Karachi 74800, Pakistan; 3 Department of Radiology, Aga Khan University Hospital, Stadium Road, PO BOX: 3500, Karachi 74800, Pakistan

**Keywords:** Ewing’s sarcoma, Adrenal, CD99

## Abstract

**Background:**

Ewing’s sarcoma uncommonly arises from extraosseous soft tissue or parenchymal organs. Primary adrenal Ewing’s Sarcoma, although very rare, is extremely aggressive and commonly fatal.

**Case presentation:**

A 17 year old Pakistani male was referred to the outpatient oncology clinic at our center with a three month history of concomitant pain, swelling and dragging sensation in the right hypochondrium. Abdominal examination revealed a large, firm mass in the right hypochondrium extending into the right lumbar region and epigastrium. His genital exam was unremarkable and there were no stigmata of hepatic or adrenal disease.

Computed tomography scans revealed a large peripherally enhancing mass in the hepatorenal area, biopsy of which showed a neoplastic lesion composed of small round blue cells which exhibited abundance of glycogen and stained diffusely positive for CD99 (MIC2 antigen). Fluorescence in situ hybridization demonstrated gene rearrangement at chromosome 22q12 which confirmed the diagnosis of Ewing’s sarcoma. Staging scans revealed pulmonary metastasis and hence he was commenced on systemic chemotherapy.

**Conclusion:**

This case report highlights the importance of keeping Ewing’s sarcoma in mind when a young patient presents with a large non-functional adrenal mass.

## Background

Ewing sarcoma (ES) and peripheral primitive neuroectodermal tumor (PNET) are part of a spectrum of diseases comprising the Ewing sarcoma family of tumors (ESFTs) which most commonly arise from long and flat bones and share similar histologic and immunohistochemical characteristics [1]. ESFTs characteristically express CD99 (Mic2 antigen) and the defining characteristic translocation is t(11;22)(q24;q12) [1].

Although ES/PNET most commonly develops in bone and soft tissues, solid organ primaries have been reported in the past at locations including the paravertebral areas and along the genitourinary tract [2]. The adrenal gland has very rarely been implicated as the primary site of ESFTs after excluding osseous disease but has been documented to be an extremely aggressive and lethal disease when it does occur [3-8]. ES/PNET arising from the adrenal gland has the potential of being misdiagnosed as a neuroblastoma with which it shares the morphology of small round blue cell tumor [9].

We report the case of a young man with ES/PNET of the adrenal gland. We believe that this is the first reported case of this rare presentation from our center and possibly from the country. The case highlights the importance of having ESFTs in the differential diagnosis of a non-functional adrenal mass in order to make a correct diagnosis, as the management and prognosis of ESFTs is entirely different from other possible causes.

## Case presentation

A 17 year old Pakistani male was referred to the outpatient oncology clinic at our center with a three month history of swelling in the right upper abdomen with concomitant pain and dragging sensation in the right hypochondrium. An abdominal ultrasonogram ordered by his referring physician had revealed a huge retroperitoneal soft tissue mass of unclear origin.

He was uncomfortable due to moderate pain at his first clinic visit and though vitally stable, was obviously pale. In the absence of icterus and pedal edema, abdominal examination revealed a large, firm mass in the right hypochondrium extending into the right lumbar region and epigastrium. His genital exam was unremarkable and there were no stigmata of chronic liver disease or Cushing’s syndrome.

**Figure 1:**
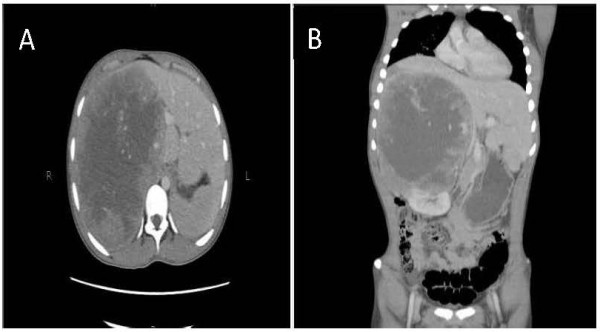
**Computed tomography scan of the abdomen at presentation.** Large mass arising from the right adrenal gland visible in axial section **(A)** and coronal section **(B)**.

Considering his age and presentation, a diagnosis of germ cell tumor was still pursued but relevant tumor markers were non-contributory although serum lactate dehydrogenase (LDH) was mildly elevated (714 IU/L). Computed tomography (CT) scans revealed a large peripherally enhancing and centrally necrotic hypervascular lesion in the hepatorenal area, arising from the right adrenal gland (Figure 1). This mass measured approximately 18.7 × 15.1 × 21.3 cm in anteroposterior, transverse and craniocaudal dimensions, was infiltrating the liver and was also causing displacement of adjacent structures to the contralateral side.

**Figure 2:**
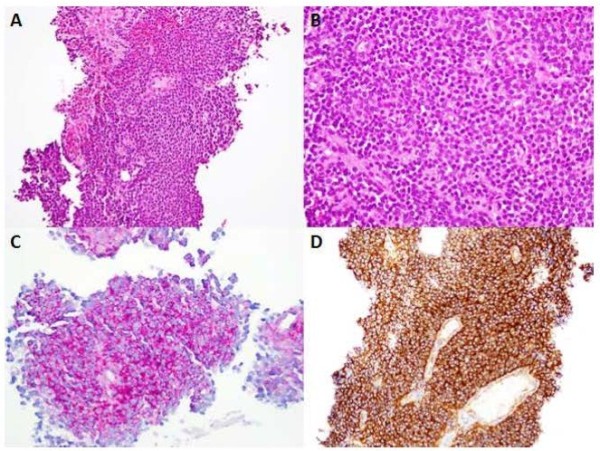
**Photomicrographs of the adrenal neoplasm.** Sheets of small rounded uniform cells with scant cytoplasm on H&E staining, as seen at a magnification of 20X **(A)** & 40X **(B)**. Tumor cells contain abundant cytoplasmic glycogen as demonstrated on PAS stain **(C)** and show strong diffuse membrane reactivity with CD99 **(D)** on immunohistochemistry.

**Figure 3:**
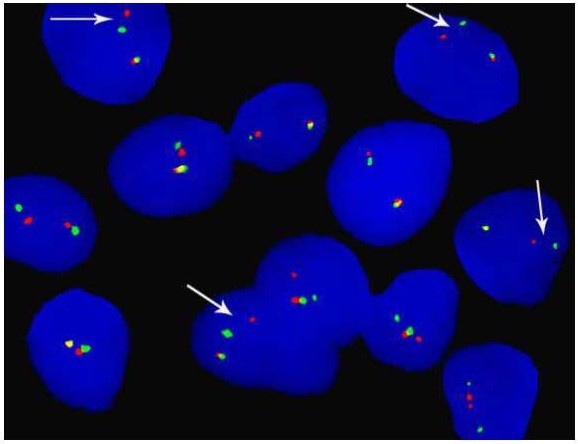
Ewing sarcoma region 1 (EWSR1) gene rearrangement at chromosome 22q12 was confirmed by Fluorescence in situ hybridization (FISH).

In view of an adrenal mass in the absence of related symptomatology, a CT guided trucut biopsy of the mass was performed. Sections from the biopsy revealed multiple tiny fragments of tissue showing a neoplastic lesion arranged in sheets with monomorphic small tumor cells containing abundant cytoplasm and demonstrating rounded nuclear contours (Figure 2A and 2B). These cells exhibited abundance of glycogen as highlighted by the periodic acid-Schiff (PAS) stain (Figure 2C) and stained diffusely positive for CD99 (MIC2 antigen) (Figure 2D). Fluorescence in situ hybridization (FISH) confirmed Ewing sarcoma region 1 (EWSR1) gene rearrangement at chromosome 22q12 (Figure 3) and hence a final diagnosis of ES/PNET was made. A bone scan was performed which ruled out osseous disease.

Staging workup unfortunately revealed multiple, nodular metastatic pulmonary deposits. He was subsequently commenced on chemotherapy following cardiac function assessment and has now completed seven months of systemic chemotherapy with alternating cycles of vincristine, doxorubicin and cyclophosphamide (VAC) and ifosfamide and etoposide (IE). He has shown promising clinical response to systemic treatment with a significant reduction in the size of the mass and alleviation of symptoms.

## Conclusions

ES/PNET occurs more commonly in adolescents with a peak incidence in the second decade of life, has a slight male preponderance [10], and typically involves the extremities and the axial skeleton [2]. Less commonly it arises in soft tissue (extraosseous Ewing sarcoma, EES) and rarely in parenchymal organs. Only a few cases of primary adrenal ES/PNET have been reported in literature [3-8]. Such is the rarity of the diagnosis that the possibility is usually not even considered in the differential diagnosis of an adrenal mass. The same holds true for our patient whereby more common diagnoses were being contemplated and the patient underwent staging workup for ES/PNET only once the biopsy results became available.

Metastatic ES/PNET needs to be considered before a conclusive diagnosis of primary adrenal disease is made. Extensive radiological imaging failed to demonstrate any other site of tumor in our patient’s case and other causes of small round blue cell tumors were excluded by an appropriate panel of immunohistochemical stains.

Despite the fact that less than a quarter of patients have overt metastasis at presentation, ESFTs is postulated to be a systemic disease. Relapse rates are as high as 80-90% in patients undergoing local therapy alone and the majority of patients now receive chemotherapy, usually administered prior to and following local treatment [11]. Multimodality treatment has infact been shown to be curative even in patients with advanced disease, although the long-term survival rates are poorer than for localized disease [11]. Following the same principle, our patient was commenced on and has now completed seven months of systemic chemotherapy.

The reporting of this case is of paramount importance because it adds to the limited number of documented cases of adrenal ES/PNET in literature and emphasizes upon the consideration of this rare diagnosis when a young patient presents with a large non-functional adrenal mass. It is true that without a high index of suspicion, the diagnosis can easily be missed.

## Consent

Written informed consent was obtained from the patient’s parent for publication of this case report and any accompanying images. A copy of the written consent is available for review by the Editor-in-Chief of this journal.

## Competing interests

The authors declare that they have no competing interests.

## Authors’ contributions

MNZ did the literature search and drafted the manuscript. TZA conceived the case report and helped in drafting the manuscript. TM and WM provided the molecular images and the radiological images respectively and provided guidance for the drafting of the manuscript. SP participated in the design and co-ordination and helped draft key areas of the manuscript in addition to providing the histopathological images. All authors read and approved the final manuscript.

## References

[B1] de Alava E Gerald WL Molecular biology of the Ewing's sarcoma/primitive neuroectodermal tumor family J Clin Oncol 2000 18 1 204 213 10623711 10.1200/JCO.2000.18.1.204

[B2] Zagar TM Triche TJ Kinsella TJ Extraosseous Ewing's sarcoma: 25 years later J Clin Oncol 2008 26 26 4230 4232 10.1200/JCO.2008.16.5308 18779607

[B3] Ahmed AA Nava VE Pham T Taubenberger JK Lichy JH Sorbara L Ewing sarcoma family of tumors in unusual sites: confirmation by rt-PCR Pediatr Dev Pathol 2006 9 6 488 495 10.2350/06-01-0007.1 17163788

[B4] Gonin J Larousserie F Dousset B Rousseau J Delattre O Waintrop C An unusual adrenal tumor: Ewing tumor Ann Pathol 2011 31 1 28 31 10.1016/j.annpat.2010.07.043 21349385

[B5] Kato K Kato Y Ijiri R Misugi K Nanba I Nagai J Ewing's sarcoma family of tumor arising in the adrenal gland–possible diagnostic pitfall in pediatric pathology: histologic, immunohistochemical, ultrastructural, and molecular study Hum Pathol 2001 32 9 1012 1016 10.1053/hupa.2001.27121 11567233

[B6] Matsuoka Y Fujii Y Akashi T Gosehi N Kihara K Primitive neuroectodermal tumour of the adrenal gland BJU Int 1999 83 4 515 516 10210585 10.1046/j.1464-410x.1999.00040.x

[B7] Pirani JF Woolums CS Dishop MK Herman JR Primitive neuroectodermal tumor of the adrenal gland J Urol 2000 163 6 1855 1856 10.1016/S0022-5347(05)67559-3 10799198

[B8] Zhang Y Li H Primitive neuroectodermal tumors of adrenal gland Jpn J Clin Oncol 2010 40 8 800 804 10.1093/jjco/hyq050 20430773

[B9] Kushner BH Cheung NK Neuroblastoma–from genetic profiles to clinical challenge N Engl J Med 2005 353 21 2215 2217 10.1056/NEJMp058251 16306518

[B10] Marina NM Etcubanas E Parham DM Bowman LC Green A Peripheral primitive neuroectodermal tumor (peripheral neuroepithelioma) in children. A review of the St. Jude experience and controversies in diagnosis and management Cancer 1989 64 9 1952 1960 10.1002/1097-0142(19891101)64:9<1952::AID-CNCR2820640931>3.0.CO;2-W 2551479

[B11] Balamuth NJ Womer RB Ewing's sarcoma Lancet Oncol 2010 11 2 184 192 10.1016/S1470-2045(09)70286-4 20152770

